# The direct and indirect association of cervical microbiota with the risk of cervical intraepithelial neoplasia

**DOI:** 10.1002/cam4.1471

**Published:** 2018-04-02

**Authors:** Chaoting Zhang, Ying Liu, Weijiao Gao, Yaqi Pan, Yunong Gao, Jing Shen, Hongchao Xiong

**Affiliations:** ^1^ Key Laboratory of Carcinogenesis and Translational Research (Ministry of Education/Beijing) Laboratory of Biochemistry and Molecular Biology Peking University Cancer Hospital & Institute Beijing 100142 China; ^2^ Key Laboratory of Carcinogenesis and Translational Research (Ministry of Education/Beijing) Laboratory of Genetics Peking University Cancer Hospital & Institute Beijing 100142 China; ^3^ Key Laboratory of Carcinogenesis and Translational Research (Ministry of Education/Beijing) Department of Gynecologic Oncology Peking University Cancer Hospital & Institute Beijing 100142 China; ^4^ Key Laboratory of Carcinogenesis and Translational Research (Ministry of Education/Beijing) Core Laboratory Peking University Cancer Hospital & Institute Beijing 100142 China; ^5^ Key Laboratory of Carcinogenesis and Translational Research (Ministry of Education/Beijing) Department of Thoracic Surgery Peking University Cancer Hospital & Institute Beijing 100142 China

**Keywords:** 16sRNA, cervical intraepithelial neoplasia, cervical microbiota, HPV, next‐generation sequencing

## Abstract

Cervical microbiota composition is associated with cervical HPV infection and CIN severity. Previous studies only assessed the total association between cervical microbiota and HPV infections or CINs, and yet no study reported the direct and indirect associations between cervical microbiota and CINs mediated by HPV infection, respectively. The aim of this study was to investigate the direct and indirect associations between microbiotas and CIN severity. Cervical microbiota of 126 women with CIN 1− (normal cytology and CIN 1) and 40 with CIN 2+ (CIN 2 and CIN 3) were analyzed using Illumina sequencing based on the 16S rRNA gene. HPV was detected using a highly sensitive PCR primer set (SPF1/GP6+). Indirect effects of *Pseudomonas stutzeri*,* Bacteroides fragilis*,* Lactobacillus delbrueckii*,* Atopobium vaginae*, and *Streptococcus agalactiae* mediated by HPV infection on CIN status were observed. The directions of the direct and the indirect associations between CIN status and *Ps. stutzeri* were opposite. The directions of the direct and the indirect associations between CIN status and *A. vaginae* were the same. *B. fragilis*,* L. delbrueckii*, and *S. agalactiae* only had indirect association with CIN status. In summary, our study provided suggestive evidence that some microbial populations could have direct or indirect effects mediated by affecting HPV infection on CIN progression. Besides HPV infection, microbial community composition possibly plays a role in cervical carcinogenesis.

## Introduction

Human papillomavirus (HPV) infection has been recognized as an important cause of cervical precancerous lesions or cancer, and yet is necessary but not sufficient for cervical carcinogenesis process 1, 2. Therefore, in addition to HPV infection, there are other factors contributing to the cervical carcinogenesis process.

**Figure 1:**
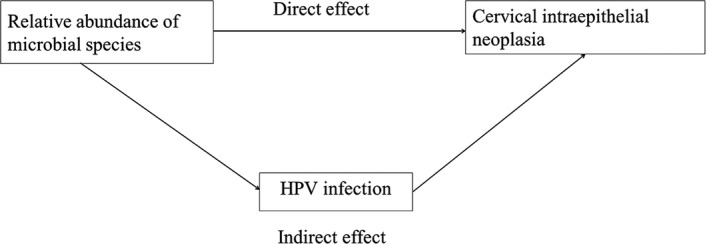
Two possible paths to explain the association between CIN status and each microbial species or community types. CIN, cervical intraepithelial neoplasia.

Some studies have demonstrated that cervical microbiota composition was associated with the acquisition, reactivation, or delayed clearance of cervical HPV infection and even CIN severity 3, 4, 5, 6, suggesting a possible role of microbial community composition in cervical carcinogenesis through potentiation of HPV infection. Therefore, the total effect of cervical microbiota composition on cervical carcinogenesis process could be explained by microbiota affecting natural history of cervical HPV infection (indirect effect) 7 and variation of microbiota composition directly affecting cervical carcinogenesis process (direct effect) 5. However, to date, all studies only assessed the total association between cervical microbiota and HPV infections or CINs, and yet no study reported the direct and indirect associations between cervical microbiota and CINs mediated by HPV infection, respectively (Fig. 1). The discovery of indirect effect mediated by microbiota affecting natural history of cervical HPV infection would give an important hint on microbiota composition affecting cervical carcinogenesis process. Therefore, we applied the appropriate analytic method in 166 individuals with CINs or normal cervical epithelium in order to decompose the direct and indirect associations between cervical microbiota and CINs.

In this study, we enrolled participants with CIN and normal cervical epithelium in order to investigate the direct and indirect associations between microbiotas and CIN severity.

## Materials and Methods

### Study population and specimen collection

A total of 166 cervical biopsy specimens were collected and diagnosed with CIN 1− (including normal cervical epithelium [*n* = 64], CIN 1 [*n* = 62]) and CIN 2+ (including CIN 2 [*n* = 19] and CIN 3 [*n* = 21]) from Beijing Cancer Hospital, Beijing, China, between 2014 and 2015. All biopsy specimens were reviewed by two experienced pathologists who confirmed the diagnosis of CIN. Individual informed consents had been collected from all participants. This study received ethical approval from the Institutional Review Board of the Peking University School of Oncology, China, and the study was carried out in accordance with approved guidelines.

The specimens were stored at −80°C and genomic DNA was extracted from the frozen tissues using DNeasy Blood & Tissue Kit (Qiagen, Hilden, Germany) following the manufacturer's instructions. All 166 specimens were used to determine microbiota composition and the 36 HPV‐positive specimens were used to identify HPV integrations.

### HPV typing

HPV DNA was detected using a highly sensitive PCR primer set (SPF1/GP6+) amplifying a 184‐bp fragment of the L1 open reading frame 8. Specimens showing the PCR amplification product were used to identify HPV genotypes. HPV probes were designed according to the full‐length genome of 17 HPV types (6, 11, 16, 18, 31, 33, 35, 39, 45, 52, 56, 58, 59, 66, 68, 69, and 82) by MyGenostics (MyGenostics, Baltimore, MD). Details of HPV typing were described previously 9.

### Overall procedure of microbiota detection

#### Amplicon generation and library preparation

PCR was used with unique barcoded primers to amplify the variable region V3–V4 of the 16S rRNA gene to create an amplicon library from cervical samples 10.

#### DNA sequencing and bioinformatic processing

The amplicon library was sequenced on an Illumina HiSeq 2500 platform (Illumina, Inc., San Diego, CA) and 250 bp paired‐end reads were generated. The raw sequences were sorted using their unique barcodes, truncated by cutting off the barcode and primer sequence, and low‐quality reads were removed 11. Sequence analysis was performed by UPARSE software 12. Sequences with ≥97% similarity were assigned to the same operational taxonomical unit (OTU). Representative sequence for each OTU was screened for further annotation. For each representative sequence, the GreenGene Database was used based on RDP classifier algorithm to annotate taxonomic information, and Lactobacillus species level was further identified using the highest pairwise similarity among the BLASTN results 13. OTUs abundance information was normalized using a standard of sequence number corresponding to the sample with the least sequences. Alpha diversity and beta diversity were calculated in QIIME using the normalized data.

### Statistical analysis

Alpha diversity metrics (e.g., Shannon index) according to CIN status were evaluated by Wilcoxon rank sum test. Principal coordinates analysis (PCoA) of weighted UniFrac metrics (beta diversity metrics) were used to visualize difference in microbial community according to CIN status. Hierarchical clustering (Euclidean distance, ward.D2 linkage) was performed based on the 18 most abundant species. Linear discriminate analysis effect size (LEfSe) was used to detect the differences in the 18 most abundant species according to the HPV infection and CIN status using the default values of *α* = 0.05 and LDA = 2.0 14. For further evaluation, each microbe was categorized into three groups (low, middle, and high) according to the p25 and p75 quartiles of each microbe among the 166 participants. Total association between CIN status and each microbial taxon or community types was decomposed into two components: direct effects and indirect effects (mediated by HPV infection as shown in Fig. 1) using ldecomp package from Stata 15. In our case, we applied the Buis procedure to a logit model, in which the outcome was the log (odds) of CIN status, exposure was percent of each microbiota (categorized into three groups), and the intermediate variable was the HPV infection status 15. As all 18 microbial species were evaluated for associations with CIN status, odds ratios (ORs) with 95% confidence intervals (CIs) were adjusted for multiple comparisons, using a bootstrap method with 1000 resamples 16.

*P* values reported were two‐sided and considered significant if they were <0.05. Statistical analyses were conducted using Stata 11.0 (StataCorp LP., College Station, TX).

### Accession number

Raw sequencing data were submitted to the Sequence Read Archive (study accession number PRJNA319915).

## Result

### Distribution of HPV types

As shown in Table S1, we found the prevalence of HPV infection in normal cytology, CIN1, CIN2, and CIN3 were 10.9% (7/64), 12.9% (8/62), 31.6% (6/19) and 71.4% (15/21), respectively. Of the 126 CIN 1− and 40 CIN 2+ samples, we detected HPV in 15 (11.9%) and 21 (52.5%) samples, respectively. Of the 15 HPV‐positive CIN 1− samples, we detected HPV16 (*n* = 8), HPV18 (*n* = 2), and HPV58 (*n* = 3); two samples harbored two types of HPV (HPV18 and HPV33; HPV45 and HPV58). Of the 21 HPV‐positive CIN 2+ samples, we detected HPV16 (*n* = 15), HPV33 (*n* = 2), and HPV58 (*n* = 2); two samples harbored two types of HPV (HPV52 and HPV58; HPV52 and HPV82).

### Cervical microbiota community types

**Figure 2:**
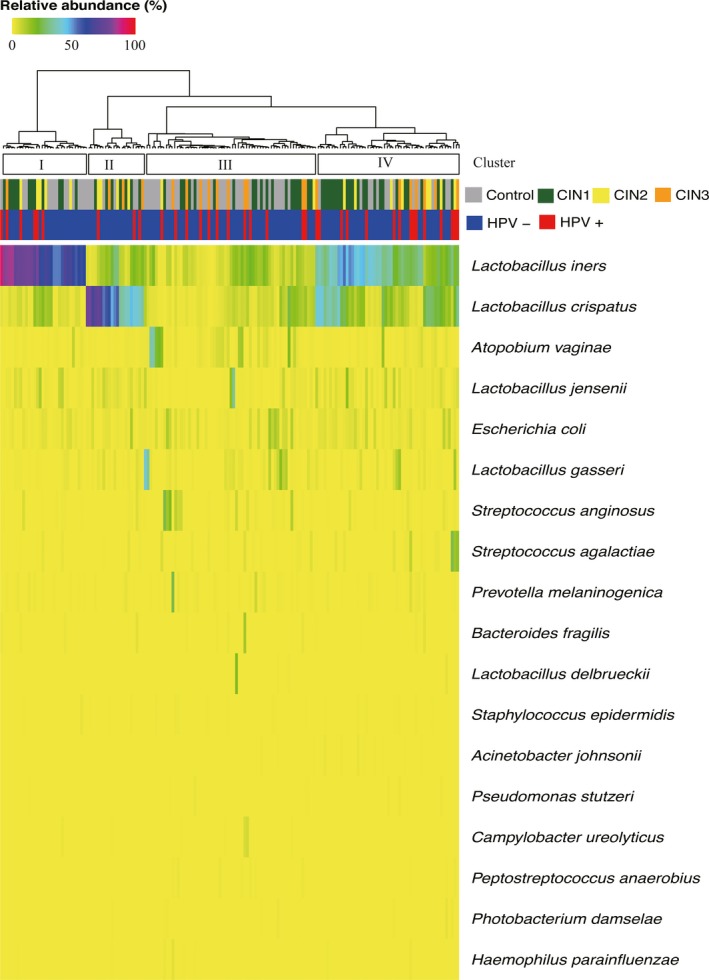
Heatmap of relative abundance of cervical microbiota at the species level among the 166 participants with normal cytology or CINs. Each vertical line represents a cervical biopsy sample. Hierarchical clustering (Euclidean distance, ward.D2 linkage) was performed based on the 18 most abundant species. HPV infection and CIN status are shown across the top of the figure. CIN, cervical intraepithelial neoplasia; HPV, human papillomavirus.

In total, 5,798,846 high‐quality gene sequences were obtained and an average of 465 OTUs per sample was detected. Overall, *Firmicutes, Actinobacteria*, and *Proteobacteria* were the three dominant phyla (Fig. S1). *Lactobacillus* and *Gardnerella* were the two most prevalent genera (Fig. S2). Cervical microbiotas were distributed into four clusters at the species level in order to visualize microbiota distribution among all individuals (Fig. 2). *Lactobacillus iners* and *L. crispatus* were dominant in cluster I and II, respectively. Cluster III contained various dominant microbes and the relative abundance of *Atopobium vaginae, Escherichia coli, Streptococcus agalactiae*, and some other microbes were higher than in other clusters (Fig. S3). *L. iners* and *L. crispatus* were the dominant members in cluster IV. The four clusters were not clearly separated by HPV infection status or histology characteristics. Additionally, we did not find an indirect association between microbiota community type and CIN status (Table S2).

### Direct and indirect associations between cervical microbiota and CIN status

**Figure 3:**
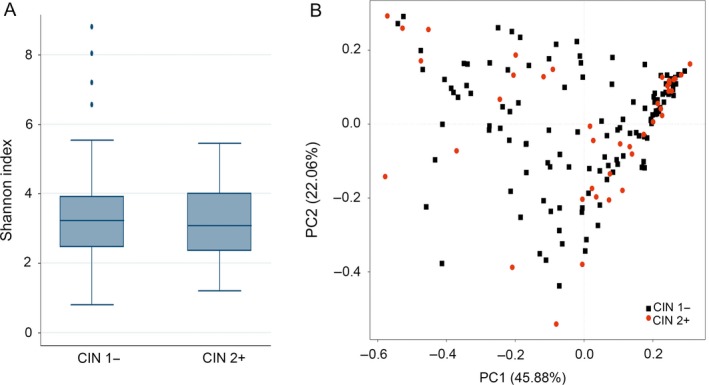
Comparison of alpha diversity metrics and beta diversity distance between CIN 1− and CIN 2+ groups. (A) Comparison of Shannon index between CIN 1− and CIN 2+ groups is shown using boxplot. (B) Ordination of the principal coordinates analysis performed on the weighted UniFrac metric. CIN, cervical intraepithelial neoplasia.

Alpha diversity did not significantly differ between CIN 1− and CIN 2+ groups (Fig. 3A). PCoA of weighted UniFrac metrics did not support difference between CIN 1− and CIN 2+ groups in microbiota community structure (Fig. 3B). Similar findings were obtained when CIN 3 was compared with normal cervical epithelium (data not shown).

**Figure 4:**
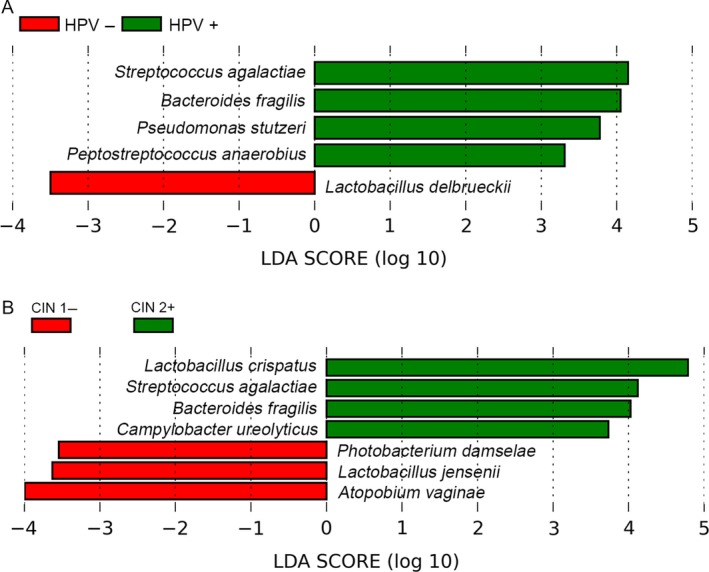
Linear discriminate analysis effect size (LEfSe) showing the differences in the 18 most abundant species according to HPV infection and CIN status. (A) LEfSe was used to detect difference in microbial relative abundance according to HPV infection. (B) LEfSe was used to detect difference in microbial relative abundance according to CIN status. CIN, cervical intraepithelial neoplasia; HPV, human papillomavirus.

LEfSe analyses evaluating microbial species discriminating on HPV infection or CIN status are shown in Figure 4. HPV‐positive samples had more *S. agalactiae*,* Bacteroides fragilis*,* Pseudomonas stutzeri*, and *Peptostreptococcus anaerobius*. The abundance of *Lactobacillus delbrueckii* was higher in HPV‐negative samples (effect size >2; *P *<* *0.05). *L. crispatus*,* S. agalactiae*,* B. fragilis*, and *Campylobacter ureolyticus* were more abundant in samples obtained from CIN 2+ cases. Higher abundance of *Photobacterium damselae*,* L. jensenii*, and *A. vaginae* was detected in CIN 1− samples.

To further evaluate the direct and indirect effect of cervical microbiota on CIN status, we categorized each microbiota into three groups (low, middle, and high) according to the p25 and p75 quartiles of each microbiota. Table 1 shows the direct and indirect associations between cervical microbiota and the CIN status based on the 18 most abundant species, and only microbiota having significantly indirect effect on CIN status are listed. Indirect effects of *Ps. stutzeri*,* B. fragilis*,* L. delbrueckii*,* A. vaginae*, and *S. agalactiae* mediated by HPV infection on CIN status were observed. The directions of the direct and the indirect associations between CIN status and *Ps. stutzeri* were opposite. The directions of the direct and the indirect associations between CIN status and *A. vaginae* were the same. *B. fragilis*,* L. delbrueckii*, and *S. agalactiae* only had indirect association with CIN status.

**Table 1:** Direct and indirect associations between microbial species and cervical intraepithelial neoplasia (CIN) among 166 women in China

Variable	CIN1−a, *n*	CIN2+^a^, *n*	Direct OR, (95% CI)^b^	Indirect OR, (95% CI)^b^
*Pseudomonas stutzeri*				
Low	27	14	Ref.	Ref.
Middle	68	16	**0.39 (0.17, 0.90)**	1.17 (0.86, 1.61)
High	31	10	**0.36 (0.15, 0.85)**	**1.73 (1.11, 2.72)**
*Bacteroides fragilis*				
Low	102	27	Ref.	Ref.
Middle	0	0	NA	NA
High	24	13	1.11 (0.57, 2.17)	**1.84 (1.18, 2.85)**
*Lactobacillus delbrueckii*				
Low	59	17	Ref.	Ref.
Middle	32	17	1.72 (0.80, 3.69)	1.07 (0.73, 1.57)
High	35	6	1.35 (0.37, 4.99)	**0.44 (0.24, 0.81)**
*Atopobium vaginae*				
Low	23	18	Ref.	Ref.
Middle	67	17	0.50 (0.20, 1.21)	**0.65 (0.43, 0.98)**
High	36	5	**0.28 (0.08, 0.95)**	**0.63 (0.41, 0.97)**
*Streptococcus agalactiae*				
Low	38	3	Ref.	Ref.
Middle	63	21	2.24 (0.68, 7.43)	**1.88 (1.23, 2.89)**
High	25	16	2.99 (0.84, 10.61)	**2.71 (1.45, 5.07)**

CIN, cervical intraepithelial neoplasia; OR, odds ratios; CI, confidence interval.

a CIN1− included normal cervical epithelium and CIN1; CIN2+ included CIN2 and CIN3.

b OR and 95% CI of all variables were estimated by parametric regression models adjusted for CIN status and HPV status were outcome and mediator variables, respectively.

Bold values refers to *P* < 0.05.

## Discussion

Our study found that *L. iners* and *L. crispatus* were the predominant cervical microbiota in most participating women. Some cervical microbiota not only had the direct associations with CIN severity, but also had indirect effect on CIN severity mediated by affecting HPV infection status. The directions of the direct and indirect associations between CIN status and cervical microbiota could be the same or opposite.

*Lactobacillus iners* and *Lactobacillus crispatus* were the predominant cervical microbiotas in most of the participating women. These two species were also dominant in previous studies in Asian women and yet their relative abundance in our study was lower than those reported previously 6, 17, 18. The similar findings (dominance of these two species) of different studies may be due to low diversity of cervical microbiota when compared with microbiota community from other parts of the human body. The lower abundance of these two species in our study may be due to various factors, including sample collection and sequencing methods, temporal shift in microbiota communities, as well as CIN status and HPV infection 7, 19, 20, 21, 22.

In this study, high abundance of *L. jensenii* and *L. crispatus* were found in CIN 2+ and CIN 1− samples, respectively; while we did not observe the association between *L. iners* and CIN severity. Although cervical *Lactobacillus spp*. are dominant and maintain a low pH by producing lactic acid in most women, different species could play distinctive roles in cervical carcinogenesis process 6. Besides *Lactobacillus spp*., we also found some other microbes with high abundance in CIN 1− and CIN 2+ groups, respectively. More importantly, we found indirect associations between cervical microbiotas and CINs mediated by affecting HPV infection status and moreover directions of the direct and indirect associations could be the same or opposite in different microbes, indicating that the variation of microbiota composition could directly affect cervical carcinogenesis process and with indirect effect mediated by HPV infection. Although many studies have elucidated the host‐commensal microbiota interaction in gastrointestinal tract and to some extent in pelvic inflammatory diseases and pregnancy complications 7, 17, 19, 23, to date only three studies assessed the associations between microbial communities and CIN severity 5, 6, 24. Specimens were obtained by cervical swabs in all three studies, and sampling locations were posterior vaginal fornix 24 and cervix 5, 6. As microbial community composition varies greatly in different regions of the female genital tract and using different sampling methods 19, microbial community composition of the exfoliated cells obtained from cervical swabs reflected the microbial community in the entire cervix including the CIN region and the normal cervical epithelium, and therefore could not accurately reflect the microbial community in the CIN regions, whereas in our study cervical biopsy specimens were obtained directly from the CIN regions, which represented the real microbial community of the CIN regions more accurately. In addition, these three studies only investigate the total effect of cervical microbiota composition on cervical carcinogenesis process but not evaluate the direct and indirect effect, respectively. The indirect effect of some microbiota mediated by affecting natural history of cervical HPV infection highlighted the importance of interaction between cervical microbial community and HPV infection on cervical carcinogenesis process. Moreover, some microbiota had opposite direction of direct and indirect effect on cervical carcinogenesis process, which could result into failing to find the relationship between this microbiota and cervical carcinogenesis process in previous studies only investigating total association.

However, there are some limitations in our study. First, the associations between CIN severity and the microbial community were not adjusted for other risk factors due to unavailability of demographic characteristics. Second, the cross‐sectional study did not investigate the temporal relationship between cervical microbiotas and CINs. Third, we did not clarify the mechanism of interplay between cervical microbiotas and HPV infection for cervical carcinogenesis in this study. Fourth, as sample size was small and moreover site of sampling is at the location of CIN but not entire cervix, the proportion of HPV infection in our individuals could be underestimated. Fifth, as the HPV‐positive sample size is small, we would enroll more individuals to validate our study findings in the near future. Sixth, although HPV DNA was detected using a highly sensitive PCR primer set (SPF1/GP6+) amplifying a 184‐bp fragment of the L1 open reading frame, this assay might also underestimate the proportion of some type HPV infection.

In summary, our study provided suggestive evidence that some microbial populations could have direct or indirect effects mediated by affecting HPV infection on CIN progression. Besides HPV infection, microbial community composition possibly plays a role in cervical carcinogenesis.

## Conflict of Interest

The authors have no conflict of interest.

## Supporting information


**Figure S1.** Distribution of cervical microbiota community types at the phylum level among the 166 participants.

**Figure S2.** Distribution of cervical microbiota community types at the genus level among the 166 participants.

**Figure S3.** Differences in the 18 most abundant species according to the four community types.

**Table S1**. HPV dristibution in CIN1− and CIN2+ cases^a^.
**Table S2**. The associations between cervical mucosa community type and CIN or HPV status among 166 women in China.
