# Supplementary figures and images for: The direct and indirect association of cervical microbiota with the risk of cervical intraepithelial neoplasia
Source: Cancer Med. 2018 Apr 2;7(5):2172–9. doi: 10.1002/cam4.1471 (PMC5943479; doi:10.1002/cam4.1471)

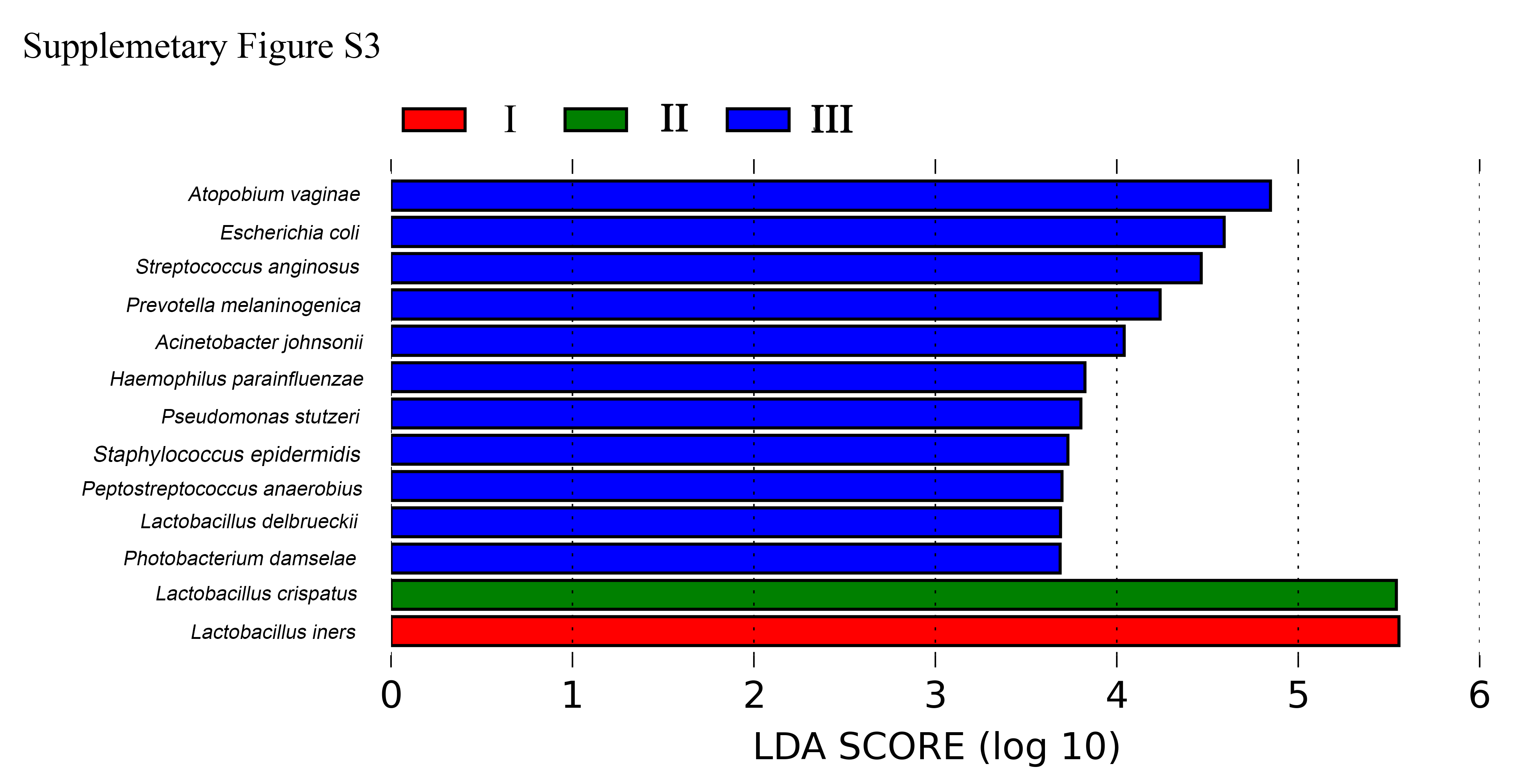

Supplement: Supplementary file 3 — Figure S3. Differences in the 18 most abundant species according to the four community types. [file CAM4-7-2172-s003.tif]
