# Supplementary material for: The direct and indirect association of cervical microbiota with the risk of cervical intraepithelial neoplasia
Source: Cancer Med. 2018 Apr 2;7(5):2172–9. doi: 10.1002/cam4.1471 (PMC5943479; doi:10.1002/cam4.1471)
Supplement: Supplementary file 4 — Table S1. HPV dristibution in CIN1− and CIN2+ casesa. Table S2. The associations between cervical mucosa community type and CIN or HPV status among 166 women in China. [file CAM4-7-2172-s004.docx]

| Supplementary Table S1. HPV dristibution in CIN1- and CIN2+ cases^a^. | | | |
| --- | --- | --- | --- |
| HPV type |  | CIN 1-(%) N=126 | CIN 2+(%) N=40 |
| HPV16 |  | 8 (6.3%) | 15 (37.5%) |
| HPV18 |  | 2 (1.6%) | 0 (0.0%) |
| HPV33 |  | 0 (0.0%) | 2 (5.0%) |
| HPV58 |  | 3 (2.4%) | 2 (5.0%) |
| HPV18 and HPV33 | | 1 (0.8%) | 0 (0.0%) |
| HPV45 and HPV58 |  | 1 (0.8%) | 0 (0.0%) |
| HPV52 and HPV58 |  | 0 (0.0%) | 1 (2.5%) |
| HPV52 and HPV82 |  | 0 (0.0%) | 1 (2.5%) |
| Abbreviations: CIN, cervical intraepithelial neoplasia; HPV, human papillomavirus. | | | |
| ^a^ CIN1- included normal cervical epithelium and CIN1; CIN2+ included CIN2 and CIN3. | | | |

| Supplementary Table S2. The associations between cervical mucosa community type and CIN or HPV status among 166 women in China. | | | | | | | | | |
| --- | --- | --- | --- | --- | --- | --- | --- | --- | --- |
| Community types | CIN 1-^a^, n | CIN 2+^a^, n | Direct OR, (95% CI)^b^ | Indirect OR, (95% CI)^b^ |  | HPV negative, n | HPV positive, n | OR,  (95% CI)^c^ |  |
| Ⅰ | 25 | 6 | Ref. | Ref. |  | 24 | 7 | Ref. |  |
| Ⅱ | 14 | 7 | 3.12 (0.63, 15.35) | 0.67 (0.32, 1.39) |  | 19 | 2 | 0.36 (0.08, 1.69) |  |
| Ⅲ | 50 | 12 | 0.96 (0.32, 2.83) | 1.04 (0.63, 1.73) |  | 47 | 15 | 1.09 (0.33, 3.59) |  |
| Ⅳ | 37 | 15 | 1.67 (0.55, 5.04) | 1.01 (0.60, 1.71) |  | 40 | 12 | 1.03 (0.31, 3.45) |  |
| Abbreviations: CIN, cervical intraepithelial neoplasia; HPV, human papillomavirus; OR, odds ratios; CI, confidence interval. | | | | | | | | |  |
| ^a^ CIN1- included normal cervical epithelium and CIN1; CIN2+ included CIN2 and CIN3.  ^b^ OR and 95% CI were estimated by parametric regression models adjusted for age, and CIN status and HPV status were outcome and mediator variables, respectively. | | | | | | | | |  |
| ^c^ ORs with 95% CIs were adjusted using a bootstrap method with 1,000 resamples. | | | | | | | | |  |
