# Supplementary material for: The direct and indirect association of cervical microbiota with the risk of cervical intraepithelial neoplasia
Source: Cancer Med. 2018 Apr 2;7(5):2172–9. doi: 10.1002/cam4.1471 (PMC5943479; doi:10.1002/cam4.1471)
Supplement: Supplementary file 5 [file CAM4-7-2172-s005.docx]

**Supplementary Figure legends**

**Supplementary Figure S1:**

Distribution of cervical microbiota community types at the phylum level among the 166 participants.

**Supplementary Figure S2:**

Distribution of cervical microbiota community types at the genus level among the 166 participants.

**Supplementary Figure S3:**

Differences in the 18 most abundant species according to the four community types.

Linear discriminate analysis effect size (LEfSe) was used to detect the differences.
